# Treatment of collagenase-induced osteoarthritis with a viral vector encoding TSG-6 results in ectopic bone formation

**DOI:** 10.7717/peerj.4771

**Published:** 2018-05-30

**Authors:** Mathijs G.A. Broeren, Irene Di Ceglie, Miranda B. Bennink, Peter L.E.M. van Lent, Wim B. van den Berg, Marije I. Koenders, Esmeralda N. Blaney Davidson, Peter M. van der Kraan, Fons A.J. van de Loo

**Affiliations:** Experimental Rheumatology, Radboud University Medical Center, Nijmegen, The Netherlands

**Keywords:** TSG-6, Osteoclasts, Gene therapy, Osteoarthritis

## Abstract

**Objective:**

Tumor necrosis factor-inducible gene 6 (TSG-6) has anti-inflammatory and chondroprotective effects in mouse models of inflammatory arthritis. Because cartilage damage and inflammation are also observed in osteoarthritis (OA), we determined the effect of viral overexpression of TSG-6 in experimental osteoarthritis.

**Methods:**

Bone marrow-derived cells were differentiated to multinucleated osteoclasts in the presence of recombinant TSG-6 or after transduction with a lentiviral TSG-6 expression vector. Multi-nucleated osteoclasts were analyzed after tartrate resistant acid phosphatase staining and resorption activity was determined on dentin slices. Collagenase-induced osteoarthritis (CIOA) was induced in C57BL/6 mice after intra-articular injection of an adenoviral TSG-6 or control luciferase expression vector. Inflammation-related protease activity was measured using bioluminescent Prosense probes. After a second adenovirus injection, cartilage damage was assessed in histological sections stained with Safranin-O. Ectopic bone formation was scored in X-ray images of the affected knees.

**Results:**

TSG-6 did not inhibit the formation of multi-nucleated osteoclasts, but caused a significant reduction in the resorption activity on dentin slices. Adenoviral TSG-6 gene therapy in CIOA could not reduce the cartilage damage compared to the luciferase control virus and no significant difference in inflammation-related protease activity was noted between the TSG-6 and control treated group. Instead, X-ray analysis and histological analysis revealed the presence of ectopic bone formation in the TSG-6 treated group.

**Conclusion:**

Gene therapy based on the expression of TSG-6 could not provide cartilage protection in experimental osteoarthritis, but instead resulted in increased ectopic bone formation.

## Introduction

No disease-modifying treatments are currently available for the treatment of osteoarthritis (OA) and affected end-stage joints are often surgically replaced (Lambova, Hermann & Muller-Ladner, 2017). OA was once viewed as mechanical wear-and-tear of the cartilage, but in recent years there is increasing evidence that OA is a complex disease affecting the whole joint (Malemud, 2015). Multiple proteins might interfere with OA pathology, including tumor necrosis factor-alpha (TNFα)-stimulated protein 6 (TSG-6) (Wisniewski & Vilcek, 2004).

TSG-6, encoded by the TNFα-induced protein 6 (TNFAIP6) gene, is glycoprotein expressed by different cell types after inflammatory stimulation (Milner & Day, 2003). While the protein is not detectable in healthy joint tissue, TSG-6 is present in the synovium, cartilage, and blood vessel walls of both rheumatoid arthritis (RA) and OA patients (Bayliss et al., 2001). In OA patients, the TSG-6 activity is associated with disease progression (Wisniewski et al., 2014). TSG-6 protein consists of a Link and a CUB domain that can bind multiple proteins and extracellular matrix molecules (Milner, Higman & Day, 2006) and has protective effects in experimental arthritis models. Transgenic mice with cartilage-specific constitutive overexpression of TSG-6 show chondroprotective, but not anti-inflammatory effects of TSG-6 in the antigen-induced arthritis model (Glant et al., 2002). In addition, recombinant TSG-6 also showed anti-inflammatory effects in collagen-induced arthritis (Mindrescu et al., 2000). Correspondingly, TSG-6 deficient mice suffered increased neutrophil influx and increased arthritis severity after induction of proteoglycan-induced arthritis (Szántó et al., 2004). Moreover, the therapeutic anti-inflammatory effects of mesenchymal stromal cells in a murine model for myocardial infarction were shown to be TSG-6-dependent (Lee et al., 2009).

Several mechanisms have been proposed for the chondroprotective and anti-inflammatory effects of TSG-6 (Milner, Higman & Day, 2006). Firstly, the Link domain of TSG-6 can bind to many cytokines and glycosaminoglycans (GAGs), which might prevent the migration of leukocytes to inflammatory sites (Dyer et al., 2014, 2016). Secondly, TSG-6 can bind non-covalently to the inter-α-inhibitor (IαI) to increase its anti-plasmin activity (Getting et al., 2002). The inhibition of plasmin may prevent the cleavage and activation of pro-matrix metalloproteinases (pro-MMPs) to protect the cartilage. In addition, TSG-6 has been shown to bind to BMP-2 and RANKL which reduced osteoclast activity, but not osteoclast differentiation (Mahoney et al., 2008, 2011).

Because cartilage damage, inflammation and bone remodeling have also been implicated in the pathogenesis of OA, we hypothesized that TSG-6 gene therapy might also have protective effects in experimental OA. In this study we first showed functionality of the TSG-6 gene therapy in vitro by inhibiting osteoclast activity. Subsequently, we tested the TSG-6 gene therapy in the collagenase-induced OA (CIOA). We did not observe therapeutic effects of TSG-6 overexpression on inflammation or cartilage damage. In contrast, we observed ectopic bone formation at the medial femur/tibia region of the joint.

## Materials and Methods

### Virus production

For cloning of the third generation self-inactivating lentiviral vector (sin), the full-length TSG-6 coding sequence was obtained from a cDNA sample of inflamed synovium of a C57BL/6 mouse by nested PCR. The first round of PCR was done by amplification using forward primer 5′-CGGCTCTGCAACCGAAGA-3′ and reverse primer 5′-ATCCAAAAGTATTTATTACAGCAAT-3′. For the second round of amplification, forward primer 5′-GTCGACGCCACCATGGTCGTCCTCC-3′ and reverse primer 5′-CATATGATCCAAAAGTATTTATTAC-3′ were used, introducing restriction sites for *Sal*I and *Nde*I, respectively, after which the TSG-6 gene was cloned in the PCR-script CAM cloning vector (Agilent Technologies, Amstelveen, Netherlands) according to the manufacturer’s protocol. Subsequently, the TSG-6 gene was cloned in the pRRL-cPPT-PGK-luc-PRE-SIN vector (PGK-TSG6) using the strategy previously described (Broeren et al., 2016). As a control vector (PGK-luc), the Trifusion reporter gene was digested from the pcDNA3.1 vector (kind gift from Robert E. Reeves, Stanford University) using *Nhe*I and *Xba*I (New England Biolabs, Ipswich, MA, USA). For adenovirus cloning, TSG-6 was digested from the PCR-script CAM vector using *Sal*I and ligated in the *Sal*I pre-digested pShuttle-CMV (Stratagene, La Jolla, CA, USA) (CMV-TSG6). The CMV-luciferase adenovirus (CMV-luc) was used as control vector. Lentiviral and adenoviral vector production were performed as described previously (Blaney Davidson et al., 2007; Broeren et al., 2016).

### Bone marrow-derived cell culture

Bone marrow-derived cells (BMDCs) of C57BL/6 mice were obtained from the femur and the tibia. Bone marrow cells were flushed from the bones using RPMI medium supplemented with 10% fetal bovine serum (FBS), 1 mM pyruvate and 1% penicillin/streptomycin (P/S) using a Microlance 3 needle (Becton Dickinson (BD), Breda, The Netherlands). Cells were passed trough a 70 μm cell strainer (Corning, NY, USA) and centrifuged for 5 min at 1,500 rpm/423 g in a Heraeus Megafuge 16R (Thermo Scientific, Waltham, MA, USA). In a 96-well plate (Greiner Bio-one, Alphen a/d Rijn, The Netherlands), 10^5^ cells/well were seeded on the surface of the plate for differentiation analysis or on an elephant dentin slice for the bone resoption assay. Osteoclast differentiation was induced in alpha-MEM medium, supplemented with 5% FBS, 1% P/S, 30 ng/ml murine M-CSF (R&D systems, Oxford, UK) and 20 ng/ml soluble murine RANKL (R&D Systems, Oxford, UK). For tartrate resistant acid phosphatase (TRAP) staining, after 72 h the the osteoclast medium was replaced to induce osteoclast fusion with or without 1 μg/ml recombinant murine TSG-6 (rmTSG-6) (R&D Systems, Oxford, UK) for 24 h. Subsequently, cells were fixed and stained with the TRAP staining kit (Sigma-Aldrich, Zwijndrecht, The Netherlands) according to the manufacturer’s protocol. For the bone resorption assay, after 72 h the differentiation medium was replaced and cells were transduced with 166 ng lentivirus per 10^5^ cells in each well or left untreated until day 4, at which an optimal dose 1 μg/ml rmTSG-6 (based on Mahoney et al., 2008) was added to the rmTSG-6 group to align with protein production in the TSG6-transduced cells. At day 7, medium was removed from all dentin slices. Subsequently, the cells were lysed with H_2_O.

### Bone resorption assay

After incubation with the osteoclasts, dentin slices were incubated with 10% NH_3_ and sonicated for 20 cycles 30 s on/off on a Bioruptor Next-gen (Diagenode, Seraing, Belgium) at “high” intensity. After washing, the slices were incubated for 10 min with 10% KAl(SO_4_)_2_ and stained with PhastGel Blue R-350 coomassie tablets (GE Healthcare, Eindhoven, The Netherlands) according to the manufacturer’s protocol. For the quantification, five pictures at 200× magnification were taken from every dentin slice using the Labovert FS (Leitz, Leica, Rijswijk, The Netherlands). The Leica application Suite (LAS) was used to analyse the average bone resorption percentage per dentin slice.

### Synovial biopsy culture

Synovial biopsies were obtained from surplus C.B-17 mice after sacrifice. Using a 3 mm biopsy punch (Stiefel, Wachtersbach, Germany), synovial explants were obtained and combined from the lateral and medial synovium. The biopsies were kept in 200 μl RPMI medium supplemented with 1 mM pyruvate and 1% P/S and 10^7^ infectious units adenovirus CMV-luc or CMV-TSG6 was added. After 2 h, 20 μl FBS was added and after 24 h, total RNA was isolated using 500 μl Tri reagent (Sigma-Aldrich, Zwijndrecht, The Netherlands) according to the manufacturer’s protocol and processed and analyzed as described below.

### RNA isolation and qPCR

Synovial biopsies in Trizol were first homogenated using Magnalyzer Green Beads (Roche Life Sciences, Almere, The Netherlands) and processed according to the provided protocol. DNA was removed by DNAse and cDNA was generated using Moloney murine leukemia virus reverse transcriptase, 0.5 μg/μl oligo(dT) primers and 12.5 mM dNTPs (Thermo Scientific). Quantative real-time PCR was perfomed as previously described (Broeren et al., 2016) using forward primer 5′-CAACCCACATGCAAAGGAG-3′ and reverse primer 5′-TACTCATTTGGGAAGCCCG-3′.

### Collagenase-induced OA

A total of 30 female C57BL/6J mice (Janvier) of 10–12 weeks old were randomized and housed in groups of five in filtertop cages at DM-II level with 12 h light-dark cycles and water and standard diet (AB Diets, Woerden, The Netherlands) were provided ad libitum. The mice received an intra-articular (i.a.) injection of 10^7^ infectious units adenovirus CMV-luc or CMV-TSG6 dissolved in 6 μl 0.9% NaCl four days prior to the start of the induction of the model. All i.a. injections were performed during the day in the right knee using a BD microlance needle 30G 1/2′ (BD) under general anaesthesia using 2.5% isoflurane. The injections were performed by a technician without knowledge of the viral vector content in a laminar flow cabinet. CIOA was induced by two i.a. injection of one unit collagenase type VII (Sigma-Aldrich, Zwijndrecht, The Netherlands) in 6 μl 0.9% NaCl at day 1 and day 3 as described previously (Schelbergen et al., 2015). At day 21, a second injection of 10^7^ infectious units adenovirus of the same viral vector was given and mice were sacrificed by cervical dislocation at day 42. All animal experiments were approved by the local authority Animal Care and Use Committee and local ethics committee (RU-DEC 2014-080). Animal care was in accordance with the institution guidelines.

### Prosense measurement

Six days after the first collagenase injection, five mice per group received i.v. injections of 1.33 nmol Prosense 680 probes (PerkinElmer, Groningen, The Netherlands) in 100 μl in the orbita plexus. At day 7, hair was removed from the knees and the mice were imaged with the IVIS Lumina (PerkinElmer, Groningen, The Netherlands) using the Cy5.5 filter. The data was analyzed using Living Image 3.0 (PerkinElmer, Groningen, The Netherlands). Regions of interest of the same size were drawn around the knees and the florescence intensity were determined for the experimental and contralateral knees.

### X-ray imaging

After sacrifice, the mouse knees were removed and imaged using the Faxitron FX-20 (Faxitron, Tucson, AZ, USA) at 26 kV for 10 s. The images were blinded and randomized and the ectopic bone formation was given an arbitrary score of 0–5. 0 = No ectopic bone formation visible, 1 = ectopic bone formation just detectable, 2 = clear ectopic bone formation, 3 = bone formation stretching along femur and tibia, 4 = large ectopic bone formation, 5 = severe ectopic bone formation, similar to the most severe sample.

### Histological analysis

After X-ray imaging, knee joints were fixed in formalin, embedded in paraffin and cut in 7 μm sections. The sections were stained with Safranin-O/Fast Green, blinded and randomized. Cartilage damage was assessed using an arbitrary score of 0–30 based on the OARSI cartilage OA histopathology grading system (Pritzker et al., 2006), modified for the assessment of murine knee joints by scoring the staging 0–5. Several mice showed dislocation of the knee joint, but these were distributed equally between the groups and are incorporated in the analysis.

### Statistical analysis

Statistical comparisons were performed by one-way analysis of variance (ANOVA) and Mann–Whitney *U* test as indicated in the text using GraphPad Prism 5.03. Results are depicted as mean ± 95% confidence interval (CI) and *p*-values below 0.05 were regarded as significant.

## Results

### TSG-6 inhibits osteoclast activity

An increase in TRAP-positive osteoclasts is observed in the subchondral bone of OA patients (Prieto-Potin et al., 2015). We therefore first tested the functionality of viral expression of TSG-6 in vitro by assessing its effects on osteoclast activity. BMDCs were differentiated to multinucleated osteoclasts using M-CSF and RANKL in the absence or presence of recombinant murine TSG-6. The formation of multi-nucleated osteoclasts from BMDCs was observed in conditions with and without TSG-6 (Fig. S1).

**Figure 1:**
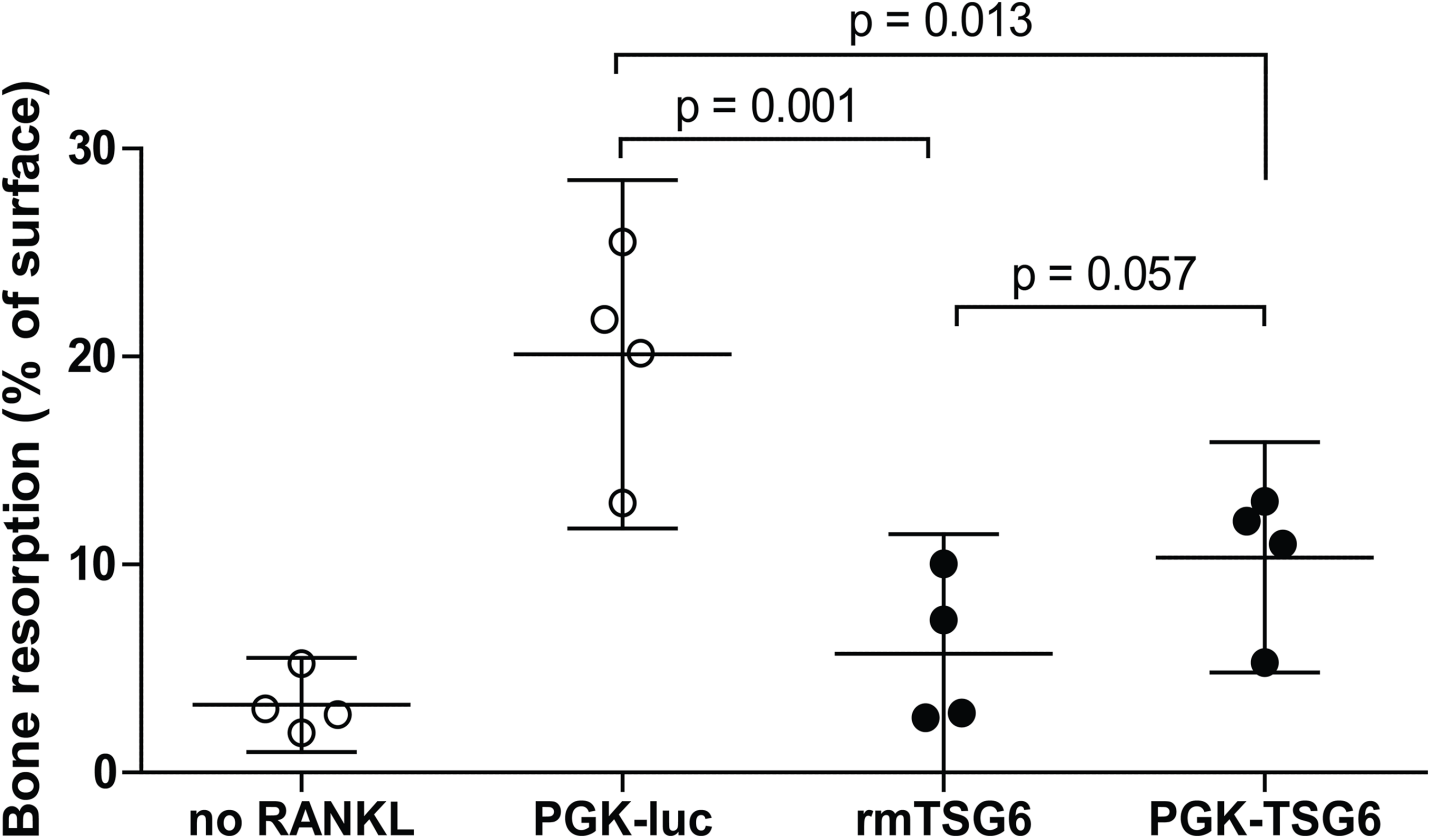
Bone resorption by bone marrow-derived cells (BMDCs) after TSG-6 treatment. BMDCs were seeded on dentin slices and differentiated to osteoclasts. After three days of differentiation, cells were transduced with control of TSG-6 virus or not transduced. After 24 h, 1 μg/ml recombinant murine TSG-6 (rmTSG6) was added to the rmTSG-6 group The cells were incubated for three additional days and thereafter the resorption pits were evaluated. RANKL was present during the whole experiment, but was omitted in the “no RANKL” group. Every sample represents an average of five pictures and the bone resorption is depicted as percentage resorption of the complete surface. The results are representative for multiple experiments and values are depicted as mean ± 95% confidence interval (CI). Statistical comparisons were performed by one-way ANOVA.

We subsequently assessed the effects of TSG-6 on osteoclast resorption activity on dentin slices. Under positive control conditions with lentiviral control virus, resorption pits covered ∼20% of the dentin surface (Fig. 1). The resorption by the multinucleated osteoclasts was significantly reduced by both recombinant TSG-6 (∼72%) and the lentiviral expression of TSG-6 (∼49%). No statistical difference was observed between the effects of recombinant TSG-6 and lentivirus-expressed TSG-6 This shows that viral overexpression of TSG-6 can result in functional levels of TSG-6 that can inhibit osteoclast activity.

**Figure 2:**
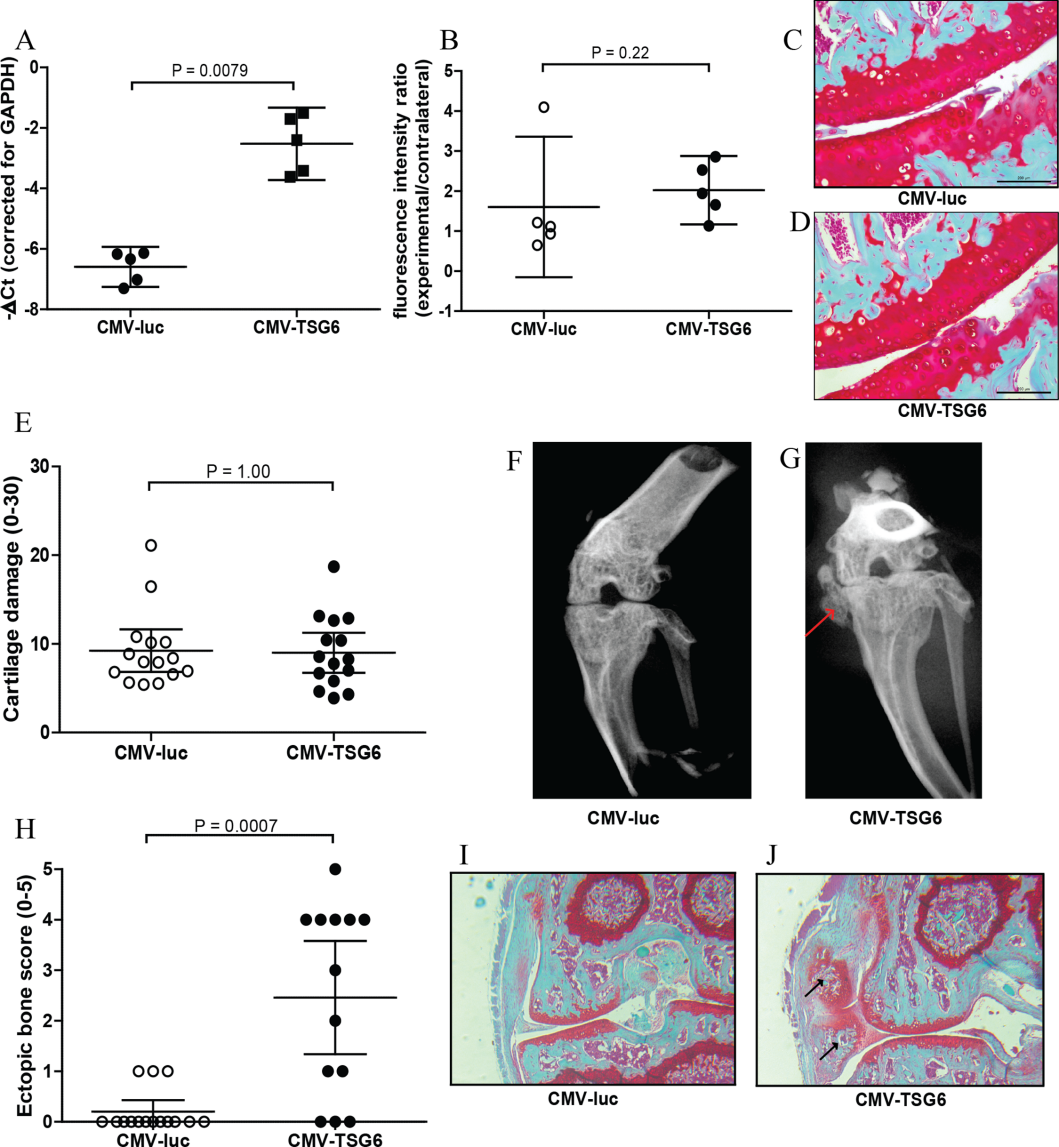
Effects of adenoviral luciferase or TSG6 on collagenase-induced osteoarthritis (CIOA) in *n* = 15 mice/group. (A) Expression of TSG-6 in synovial explants, 24 h after transduction with adenoviral CMV-luciferase (CMV-luc) or CMV-TSG6. (B) Prosense measurement at day 7 to assess the inflammation-associated protease activity in a random *n* = 5 subset/group. The fluorescence intensity ratio compared to the contralateral knee was calculated. (C, D) Magnification of examples of cartilage damage. Representative pictures of the medial femur and tibia are shown after Saphranin-O staining (average cartilage damage score 14). The scale bare indicates 200 μm. (E) Cartilage damage at day 42. For every knee joint, the cartilage damage was determined in the medal tibia, medial femur, lateral tibia, and lateral femur in three sections. The average cartilage damage is depicted. (F, G) Typical X-ray images of knee joints at 42, score 0 and 5, respectively. Ectopic bone formation is indicated by the red arrow. (H) Arbitrary scoring of ectopic bone formation (0–5) in the X-ray images. (I, J) Histological sections of the knee joints shown in Fig. 2E. Sections were stained with Safranin-O and counterstained with Fast Green. Ectopic bone formation is indicated with black arrows. Quantitative results are depicted as mean ± 95% confidence interval (CI) and statistical comparisons were performed by Mann–Whitney *U* test.

Overexpression of TSG-6 in synovium was first investigated in synovial explants. After transduction with adenoviral CMV-TSG6, the expression of TSG-6 was significantly increased compared to CMV-luciferase (Fig. 2A).

### TSG-6 effects in CIOA

The protective effects of TSG-6 on inflammation and cartilage damage were tested in the CIOA model, an experimental OA that includes inflammation (Schelbergen et al., 2015). Mice received an injection with adenovirus to provide high expression levels of TSG-6 or control luciferase in the right knee four days prior to the first collagenase injection. The effects of TSG-6 on inflammation-associated protease activity was determined in a subset of the mice after injection of Prosense 680 probes at day 6 after the first collagense injection. At day 7, the fluorescence intensity was measured and compared to the naïve contralateral knee. No significant differences in protease activity were found between the TSG-6 adenovirus (average 2.0-fold compared to contralateral joint) and the luciferase control virus (average 1.6-fold compared to contralateral joint) (Fig. 2B). A second adenovirus injection was given at day 20 to provide TSG-6 expression for the second half of the model and mice were sacrificed at day 42. The cartilage damage was assessed in histological sections using the OARSI cartilage OA histopathology grading system. No significant differences were observed in cartilage damage between the control group and the TSG-6 treated group (Figs. 2C–2E).

Before the joints were processed for histological analysis, the bone structure was analyzed using X-ray imaging and scored using an arbitrary scoring method from 0 to 5. Surprisingly, mice treated with adenoviral TSG-6 showed significantly more ectopic bone formation (77%) compared to the control group (20%) located at the medial collateral ligament (Figs. 2F–2H). Safranin-O staining of histological sections of the joints shown in Figs. 2F and 2G shows the presence of cartilage around the ectopic bone, indicating that the ectopic bone formation might be the result of endochondral ossification (Figs. 2I and 2J).

## Discussion

In this study, we show that in vitro viral overexpression of TSG-6 in BMDCs has no effect on multinuclear osteoclast formation, but can reduce resorption activity of the osteoclasts. This has been observed in earlier studies (Mahoney et al., 2008, 2011) and shows that functional TSG-6 can be expressed after viral gene transfer and reduce in vitro bone resorption. However, when tested in the CIOA model, TSG-6 could not reduce cartilage damage. In contrast, we observed increased ectopic bone formation in mice with TSG-6 overexpression.

The lack of protective effects from TSG-6 overexpression on the development of cartilage damage, despite the inhibitory effects on osteoclast activity, might be related to the indications that cartilage damage in the CIOA model is caused by mechanical stress resulting from joint instability (van der Kraan et al., 1990). Mechanical stress is implicated as an important cause for cartilage damage in many OA patients (Guilak, 2011; Buckwalter et al., 2013), but is distinct from the mechanisms that cause cartilage damage in the RA models in which TSG-6 treatment was successful.

Although TSG-6 is associated with different functions that might be protective for OA, the expression and activity of TSG-6 correlate with progression of OA (Wisniewski et al., 2014). The exact mechanisms by which TSG-6 might be involved OA progression is not completely understood and is difficult to study because of the ability of TSG-6 to bind many different proteins and matrix components. Thus, TSG-6 may have tissue-specific functions, resulting in protective effects in experimental RA, but detrimental effects in OA models. One potential mechanism could be related to a disturbance of damage repair mechanisms. TSG-6 has been shown to bind to fibronectin (FN), stimulating FN matrix assembly involved in damage repair (Kuznetsova et al., 2008). However, TSG-6 can also serve as a bridging molecule between FN and thrombospondin-1 (TSP-1). TSP-1 is increased in OA chondrocytes and osteophytes and has multiple functions, including the activation of latent transforming growth factor-β (TGF-β) (Crawford et al., 1998; Pfander et al., 2000). Increased TGF-β activity has previously been correlated with chondrocyte differentiaton and enthesophyte formation similar to this study in the medial collateral ligament in CIOA (Blaney Davidson et al., 2007; de Munter et al., 2013). The formation of osteophytes in these marginal locations is associated with OA progression in human OA (Felson et al., 2005).

An alternative mechanism for increased TGF-β activity could be related to hyaluronan (HA). In the context of fibrosis, studies have shown that the heavy chain transfer to HA by TSG-6 can stabilize the HA coat, which is essential for TGF-β-mediated myofibroblast differentiation (Meran et al., 2013). Possibly, TSG-6 can increase TGF-β-mediated chondrogenesis in a similar way. HA could also increase chondrogenesis in adipose-derived stem cells (Wu et al., 2013). This was dependent on the interaction between HA and CD44, which is influenced by TSG-6 (Lesley et al., 2004). The formation of bone is dependent on the ratio between bone matrix deposition and bone resorption. The inhibiting effects on osteoclast activity observed in Fig. 1 might favor the anabolic activity and stimulate the ectopic bone formation. Although the exact mechanism by which TSG-6 can increase ectopic bone formation in vivo remains to be elucidated, no improvement in cartilage damage in was observed, indicating that intra-articular gene therapy with TSG-6 might not be a promising treatment for OA.

## Supplemental Information

10.7717/peerj.4771/supp-1 Supplemental Information 1 Raw data.

10.7717/peerj.4771/supp-2 Supplemental Information 2 Osteoclastogenesis and bone resorption by bone marrow-derived cells (BMDCs) after TSG-6 treatment. **(A)** Representative TRAP staining of BMDCs differentiated to osteoclasts using M-CSF and RANKL. Multi-nucleated osteoclasts are indicated by black arrows. **(B)** Typical example of bone resorption on dentin slices by osteoclasts.

10.7717/peerj.4771/supp-3 Supplemental Information 3 NC3Rs ARRIVE guidelines checklist.

## Additional Information and Declarations

### Data Availability

The following information was supplied regarding data availability: The raw data are provided in a Supplemental File.
