# Supplementary figures and images for: Treatment of collagenase-induced osteoarthritis with a viral vector encoding TSG-6 results in ectopic bone formation
Source: PeerJ. 2018 May 30;6:e4771. doi: 10.7717/peerj.4771 (PMC5984587; doi:10.7717/peerj.4771)

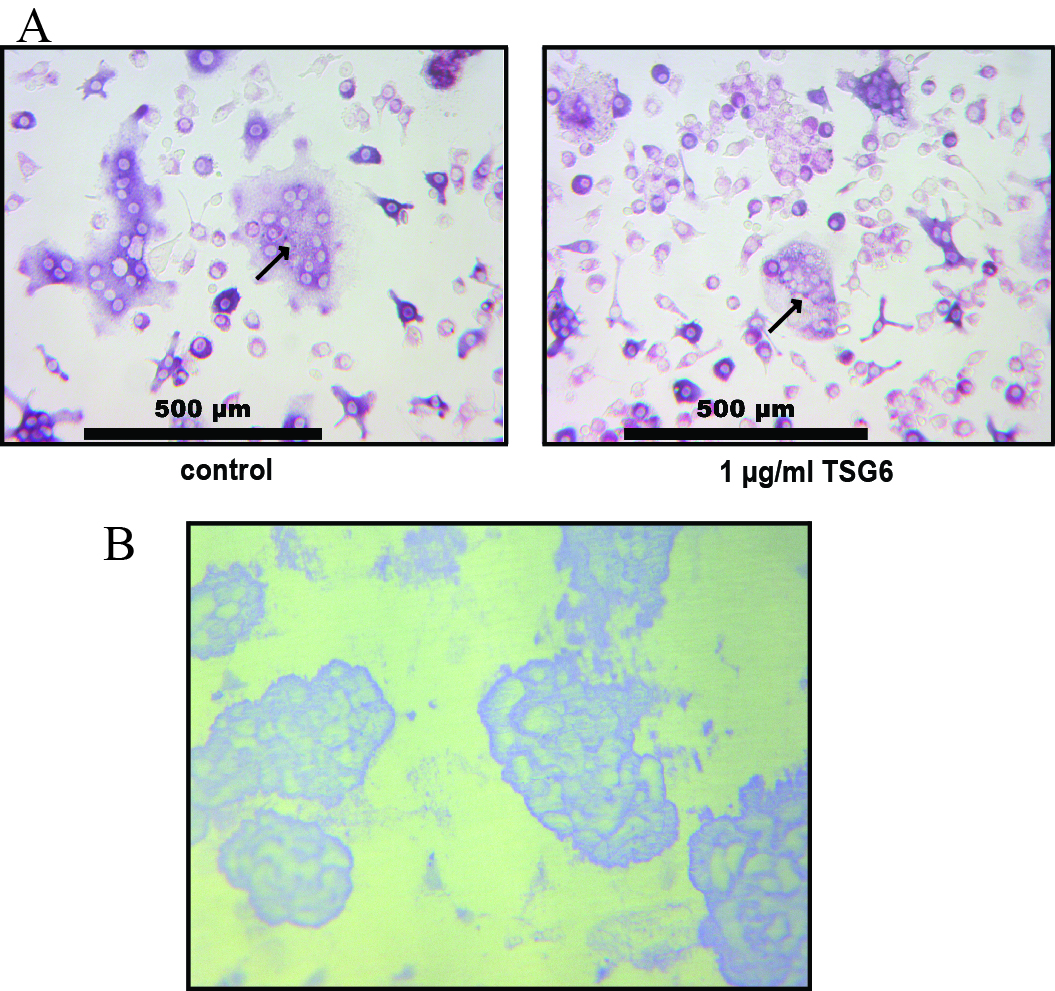

Supplement: Supplemental Information 2 — (A) Representative TRAP staining of BMDCs differentiated to osteoclasts using M-CSF and RANKL. Multi-nucleated osteoclasts are indicated by black arrows. (B) Typical example of bone resorption on dentin slices by osteoclasts. [file peerj-06-4771-s002.jpg]
